# Integrated bioinformatics analysis identifies shared immune changes between ischemic stroke and COVID 19

**DOI:** 10.3389/fimmu.2023.1102281

**Published:** 2023-03-08

**Authors:** Wenhao Liu, Fei Han, Mengyao Wan, Xin-Zhuang Yang

**Affiliations:** ^1^ Eight-year program of Clinical Medicine, Chinese Academy of Medical Sciences & Peking Union Medical College, Beijing, China; ^2^ Department of Neurology, Peking Union Medical College Hospital, Chinese Academy of Medical Sciences and Peking Union Medical College, Beijing, China; ^3^ Medical Research Center, State Key laboratory of Complex Severe and Rare Diseases, Peking Union Medical College Hospital, Chinese Academy of Medical Sciences and Peking Union Medical College, Beijing, China

**Keywords:** COVID-19, ischemic stroke, gene expression profiles, immune system, immune cell proportion

## Abstract

Although COVID-19 is primarily a respiratory disease, its neurological complications, such as ischemic stroke (IS), have aroused growing concerns and reports. However, the molecular mechanisms that underlie IS and COVID-19 are not well understood. Therefore, we implemented transcriptomic analysis from eight GEO datasets consist of 1191 samples to detect common pathways and molecular biomarkers in IS and COVID-19 that help understand the linkage between them. Differentially expressed genes (DEGs) were detected for IS and COVID-19 separately for finding shared mechanisms and we found that immune-related pathways were outlined with statistical significance. *JAK2*, which was identified as a hub gene, was supposed to be a potential therapeutic gene targets during the immunological process of COVID-19 and IS. Besides, we found a decrease in the proportion of CD8^+^ T and T helper 2 cells in the peripheral circulation of both COVID and IS patients, and *NCR3* expression was significantly correlated with this change. In conclusion, we demonstrated that transcriptomic analyses reported in this study could make a deeper understanding of the common mechanism and might be promising for effective therapeutic for IS and COVID-19.

## 1 Introduction

Although the cause of ischemic stroke associated with COVID-19 is unclear, ischemic stroke (IS) is a major contributor of morbidity and mortality in patients infected with SARS-COV2 (1–3). There is rising incidence that post-COVID-19 stroke patients tend to lack the cardiovascular risk factors (4–6). Besides, numerous independent studies have reported increasing arterial and venous thrombosis, which are probably caused by the activation of the immune system in response to viral pathogen invasion (7, 8). Multiple mechanisms associated with SARS-CoV-2 infection and the development of COVID-19 were considered to contribute to the onset of acute ischemic stroke, which include generalized hypercoagulability, dysregulated immune response leading to the cytokine-release syndrome, damage to endothelial cells leading to increased inflammation and thrombosis (9–11).

Given the significant role of the immune system as a bridge between COVID-19 and ischemic stroke, increasing research is exploring the immune molecular mechanisms interlinking the two diseases. Cytokine storm, a hyper-inflammatory response, is the likely initiating sequence of pathological thrombosis in patients with COVID-19 (3, 4). Systemic inflammatory responses, such as cytokine storms, promote changes in immune cell polarization toward more unstable phenotypes (9). The general agreement is emerging that recent bacterial and/or viral infections can be the primary triggers of acute ischemic stroke and may be related to the prothrombotic effects of inflammatory reactions (8, 12). However, this risk seems to be higher following COVID-19 (e.g., the risk of stroke was 7.6 times higher with COVID-19 compared with influenza), probably due to the disease’s unique pathophysiological alterations (5).

**Figure 1:**
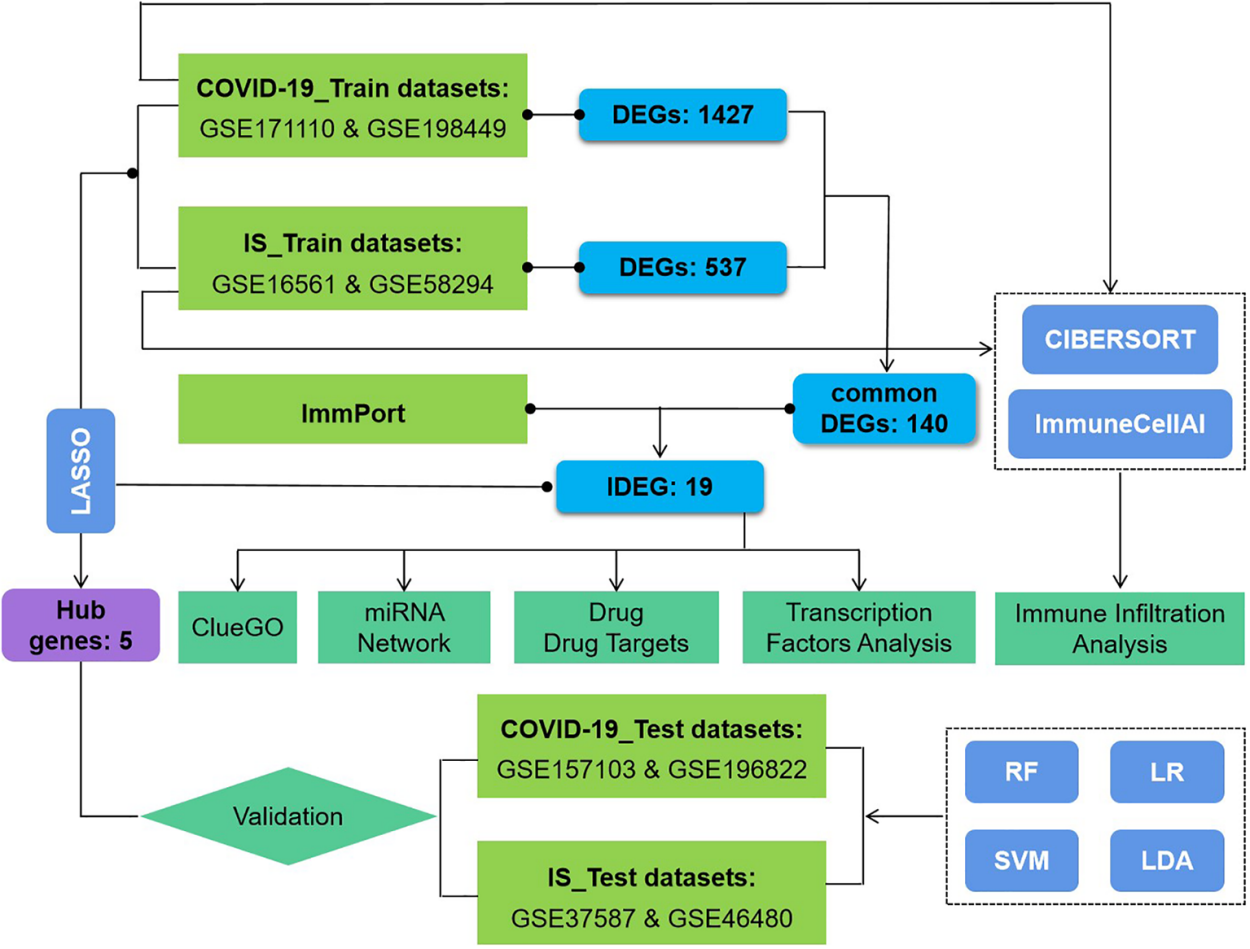
Flowchart of the study. Expression profile data from whole peripheral blood of patients with COVID-19 or ischemic stroke (IS) were obtained, and differential gene expression was performed between patients and healthy controls. ClueGO, drug targets, miRNA network, transcription factors and immune infiltration analysis were applied to common IDEGs between COVID and IS to explore shared mechanism between these two diseases. Hub genes obtained by LASSO regression were validated with test dataset of COVID and IS, using four machine learning methods. RF, Random Forest; SVM, Support Vector Machine; LR, Logistic Regression; LDA, Linear Discrimination Analysis. 108 IS patients and 47 matched controls as well as 281 COVID patients and 414 matched controls were employed to perform differential analysis.

This study used eight datasets to discover and validate the biological relationship between ischemic stroke and COVID-19. Differentially expressed genes (DEGs) were initially identified and then common DEGs of two diseases were found. Based on these common DEGs, we performed pathway analysis and confirmed the critical role of immune-related pathways. Furthermore, 19 genes were defined as immune-related among these common DEGs and further analyses of drug targets, transcript factors, and miRNA-mRNA interaction were applied on them. In addition, proportion changes of various immune cells in the peripheral blood of IS patients were evaluated using CIBERSORT and ImmunecellAI, and correlation analyses were performed between immune genes and differentially distributed immune cells. The sequential workflow of our research is presented in **Figure 1**.

## 2 Methods

### 2.1 Datasets employed in this study

The microarray datasets used in this study were obtained from the GEO database (http://www.ncbi.nlm.nih.gov/geo/). The criteria for retrieval were: A) samples were from human peripheral whole blood samples, B) gene expression was profiled, C) datasets contained both patients and healthy people without a history of stroke nor COVID-19 exposure, D) all IS patients were clinically diagnosed radiographically (with magnetic resonance imaging or computed tomography), E) all COVID-19 patients were positive for COVID-19 confirmed by RT-PCR.

To ensure the consistency and completeness of the datasets, we manually identified relevant literature using keyword filters and applied R programming language (version: 4.1.3) for subsequent analysis. Finally, IS datasets [GSE16561 (13–15) and GSE58294 (16)] and COVID-19 datasets [GSE171110 (17) and GSE198449 (18)] were included as training sets. Batch effects were corrected using the “comBat” function in the SVA package (version: 3.38.0). Next, we normalized the combined datasets and adjusted for covariates using the “Normalizebetweenarray” and “removeBatchEffect” functions in the limma package (version: 3.46.0). To validate the conclustions, we treated the GSE157103 (19) and GSE196822 (20) datasets as the validation sets for COVID-19, and GSE37587 (21), GSE46480 (22) datasets for IS which conformed to the above criteria. **Table 1** summarizes the included datasets. We also collected clinical information of COVID-19 patients from corresponding papers. Due to the diversity in description on the severity of COVID-19, we use the criteria whether patients were admitted into ICU to unify the data and avoid ambiguity (**Table S1**.)

**Table 1:** All data sets used in this study contain a total of 1191 samples, among which there were 597 cases and 594 controls.

Data sets (GEO ID)	Data		Sample type	References	Category	Phenotype	GPL
	Case	Control					
GSE16561	39	24	peripheral blood	(Barr et al., 2010; O'Connell et al., 2016; O'Connell et al., 2017) (13–15)	Train	Ischemic Stroke	GPL570
GSE58294	69	23	peripheral blood	(Stamova et al., 2014) (16)	Train	Ischemic Stroke	GPL570
GSE37587	68	0	peripheral blood	(Barr et al., 2015) (21)	Test	Ischemic Stroke	GPL6883
GSE46480	0	98	peripheral blood	(Issa et al., 2016) (22)	Test	Control	GPL570
GSE171110	44	10	peripheral blood	(Lévy et al., 2022) (23)	Train	COVID 19	GPL16791
GSE198449	237	404	peripheral blood	(Schanoski et al., 2022) (18)	Train	COVID 19	GPL24676
GSE157103	100	26	peripheral blood	(Overmyer et al., 2021) (19)	Test	COVID 19	GPL24676
GSE196822	40	9	peripheral blood	(Banerjee et al., 2022) (20)	Test	COVID 19	GPL20301

All samples were collected in the peripheral blood tissue. Control means people without COVID-19 exposure or IS, and COVID-19 exposure was defined as exposure to an individual positive for COVID-19 confirmed by RT-PCR.

### 2.2 Identification of differentially expressed genes and functional annotation

To identify differentially expressed genes (DEGs) in peripheral blood samples from COVID-19/IS patients and controls, we performed differential expression analysis using the limma package (version: 3.46.0), controlling for age and sex (24). The threshold for screening DEGs was |log_2_ FC (fold change)| > 0.5 and false discovery rate (FDR) < 0.01. Common DEGs for COVID-19 and IS were then imported to functional annotation.

Enrichment analysis of Gene Ontology (GO) and Disease Ontology (DO) was performed on common DEGs using the clusterprofiler package (version: 3.18.1) (25). Kyoto Encyclopedia of Genes and Genomes (KEGG) (http://www.genome.jp/kegg/) and gene set enrichment analysis (GSEA) were further carried out for common DEGs. The threshold for significance of the above enrichment analysis was set at FDR < 0.05. The background used for biological functional enrichment analysis were genes expressed in any samples of COVID-19 and IS in training process, respectively.

### 2.3 Hub genes and drug targets

Using immune-related genes (IRGs) downloaded from the ImmPort database, we intersected common DEGs and IRGs to generate common immune-related DEGs (IDEGs) (26). Hub genes were identified from common IDEGs using LASSO logistic regression algorithms with training datasets. The LASSO algorithm was derived from the glmnet package (version: 4.1-1) (27). LASSO logistic regression belongs to the shrinkage estimation, and during the reduction process of regression coefficients, some insignificant regression coefficients can be directly reduced to 0, that is, to the function of variable screening. We used this method on the expression matrix of DEGs of COVID and IS, respectively.

Drug and Drug_link datasets (Release Version: 5.1.9) were downloaded from the DrugBank database (https://go.drugbank.com/releases/latest) (28). The intersection of the common IDEGs and drug target genes (DTGs) was then used to generate genes targeted by drugs and potential drugs that might be promising for effective therapeutic to disease. Validation datasets were further used to examine the robustness of hub genes. Depending on hub genes’ expression, models using various data-modeling methods (random forest (RF), support vector machine (SVM), logistic regression (LR), and linear discriminant analysis (LDA)) were constructed to confirm classification performance.

### 2.4 Gene-pathway interactions and miRNA-mRNA network

To systematically explore potential biological functions between the key genes, common IDEGs were imported into the Cytoscape software v3.9.1 (https://cytoscape.org/) to construct the genes and pathways interaction network by ClueGO plug-in (29, 30). The threshold for significance of the above pathways analysis was set at P value < 0.01.

For these common IDEGs, miRNA target prediction was performed through Human microRNA Disease Database (HMDD, Version: 3.3) (31), TissueAtlas database (Current release: July 2022) (32), and Encyclopedia of RNA Interactomes (ENCORI, Version: 3.2) (33). By combined using HMDD and TissueAtlas database, we selected microRNA associated with ischemia stroke/viral infection and expressed in human blood tissue with curated experiment-supported evidence. The ENCORI website was applied to predict whether these selected miRNAs could target common IDEGs. Cytoscape software was used to visualize the miRNA-mRNA regulatory network.

### 25 Transcription factors analysis

Common IDEGs were imported into Cytospace for network analysis of transcription factors (TFs). RcisTarget package was used to acquire TFs and gene targets information, and adjusted P-value < 0.05 was considered as significant (34). Subsequently, we verified the expression levels of these TFs in training datasets of COVID-19 and IS.

### 26 Immune cell infiltration evaluation

CIBERSORT tool (version: 0.1.0) was used to generate immune cell profiles for all samples by estimating relative subsets of immune RNA transcripts (35). The CIBERSORT resulted in an expression matrix of 22 immune cells in all samples of the training dataset for COVID-19 and IS. We then used t-test to analyze the differences in immune cell components between patients and healthy controls. “ImmuCellAI” function from ImmuCellAI package (version: 0.1.0), which can accurately evaluate the abundance of immune cells, especially on multiple T-cell subpopulations (36), was applied for further analysis.

Finally, Spearman’s correlation analysis was performed between expression of common IDEGs and variation of immune cells. The ggplot2 package (version: 3.3.3) and ggpubr package (version: 0.4.0) were used to generate lollipop chart.

## 3 Results

### 3.1 Identification of common DEGs of COVID-19 and IS

To identify common pathways and molecular biomarkers shared by COVID-19 and IS on transcriptome, we initially searched the Array Express and NCBI GEO databases for expression data from whole peripheral blood of COVID-19/IS patients and healthy controls. Eight independent studies met our inclusion criteria (See Methods, **Tables 1**, **2**).

**Table 2:** Clinical characters of samples in the merged COVID training data set.

	Total sample, N(%)	COVID, N = 281(39.6%), N(%)	Control, N = 414(60.4%), N(%)	tatistics/*df*	*P* value
Gender (% female)	189 (27.2%)	96(34.2%)	93(22.5%)	X^2^ 9.6492/1	0.0019
Age,y,mean±SD	22.85±10.17	25.98±14.96	20.86±6.10	t -6.2493/693	< 0.001
Race (% white)	509(73.2%)	209(74.4%)	300(72.5%)	X^2^ 0.3127/1	0.5764

**Figure 2:**
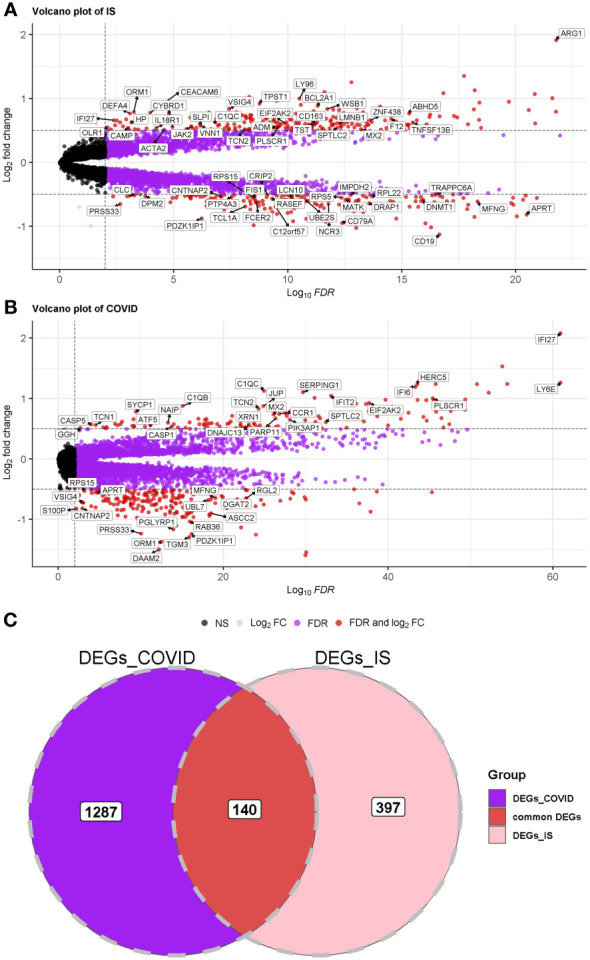
**(A, B)** Volcano plot demonstrating an overview of the differential expression of all genes in COVID-19 and IS. The threshold in the volcano plot was -log10 (adjusted P-value) > 2 and |log2 (fold change)| > 0.5; red dots indicate significant differential expressed genes. FDR was used (Benjamini Hochberg’s) for P value adjustment. **(C)** Venn diagram demonstrates the common DEGs of COVID-19 and IS.

First, we conducted an IS training dataset consisting of 108 patients and 47 matched controls by merging two IS datasets (GSE16561 and GSE58294, **Table 3**) and training dataset for COVID-19 was composed of 281 patients and 414 matched controls by combing GSE171110 and GSE198449 (**Table 3**, **Table S1**). To ensure data consistency, batch effects were controlled and the different subsets were normalized. The evaluation results showed that data pre-processing was effective and reliable **(Figure S1**). Next, differential analysis of gene expression was performed by controlling age and sex, which was significantly different between patients and healthy controls (**Tables 2**, **3**). Finally, 537 DEGs for IS and 1427 DEGs for COVID-19 were identified (See Methods, **Figures 2A, B**), and we found 140 common DEGs between COVID-19 and IS (**Figure 2C**, **Table S2**). To examine that these common DEGs were with biologically meanings, we randomly selected 537 and 1427 genes from the expressed gene sets of IS and COVID-19 separately and take interaction between them. We then repeated this operation for 1000 times and the random sampling values were significantly lower than the true observed number (T-test: P value <.001, **Figure S2**).

**Table 3:** Clinical characters of samples in the merged IS training data set.

	Total sample, N(%)	Stroke N = 108(69.7%), N(%)	Control, N = 47(30.3%), N(%)	Statistics/*df*	*P* value
Gender (% female)	80(51.6%)	55(50.9%)	25(53.2%)	X^2^ 0.0673/1	0.7953
Age,y,mean±SD	66.7±8.60	72.6±6.17	58.9±3.83	t - 13.90302/135	< 0.001
Race (% white)	126(81.3%)	84(77.8%)	42(89.3%)	X^2^ 2.88932/1	0.0892
Hypertension	93(60.0%)	70(64.8%)	23(48.9%)	X^2^ 3.44037/1	0.0636
Diabetes	30(19.4%)	23(21.3%)	7(14.9%)	X^2^ 0.8601/1	0.3537
Dyslipidemia	52(33.5%)	36(33.3%)	16(34.0%)	X^2^ 0.00739/1	0.9333

### 3.2 Functional enrichment analysis of common DEGs underlines the immune system

**Figure 3:**
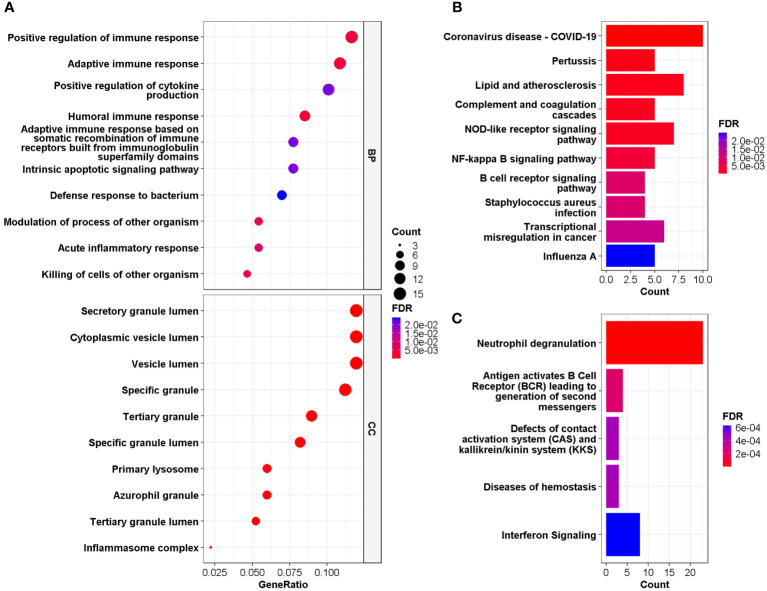
**(A)** GO enrichment analysis, where the horizontal axis represents the proportion of DEGs under the corresponding GO term. Top 10 pathways with most significant adjusted P-value were shown and ordered by gene ratio. BP, biological process; CC, cellular component. **(B, C)** GSEA of the common DEGs based on pathway database KEGG and REACTOME, where the horizontal axis represents the number of DEGs under the corresponding GSEA terms. Enrichment analysis applied Benjamini-Hochberg false discovery rate (FDR)-corrected P value.

GO and DO enrichment analyses were performed to identify the biological pathways and diseases associated with the shared DEGs. For biological processes in GO enrichment analysis, 26 pathways achieved statistical significance and 17 of them are immune-related, including positive regulation of immune response and cytokine production, adaptive immune response, humoral immune response and acute inflammatory response (**Figure 3A**). While for cellular components in GO, secretory granule lumen, cytoplasmic vesicle lumen, inflammasome complex and primary lysosome were involved (**Figure 3A**). When performing DO analysis, bronchial disease, hypersensitivity reaction type I disease, asthma, thrombocytopenia and arteriosclerosis were related to common DEGs (**Figure S3**).

GESA was further performed based on KEGG and Reactome database to decipher biological pathways behind common DEGs. The enriched molecular pathways were complement cascades, lipid and atherosclerosis, Corona Virus Disease-19, interferon and B cell receptor (BCR) signaling, neutrophil degranulation, defects of contact activation system (CAS) and kallikrein/kinin system (KKS) (**Figures 3B, C**). These results were consistent with those in GO enrichment analysis, further confirming that immune system might play essential roles in the connection between COVID-19 and IS.

### 3.3 Identification of hub genes and drug targets

**Figure 4:**
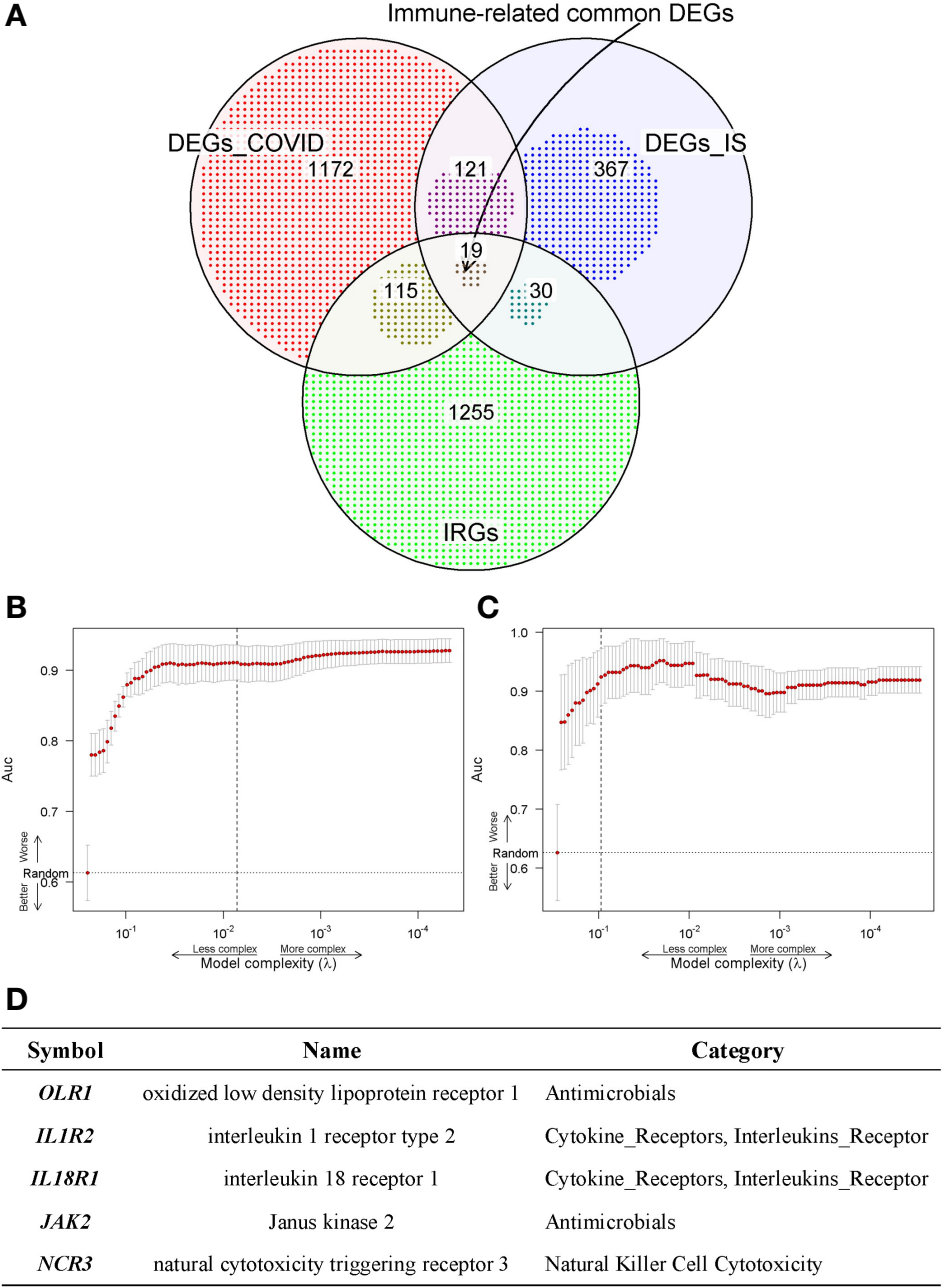
**(A)** Venn diagram shows the common IDEGs obtained by the three gene sets. **(B, C)** Construction of the key IDEGs classifier by the LASSO logistic regression algorithm shows the process of dimension reduction in COVID-19 and IS datasets, respectively. The horizontal axis represents the complexity of models, and the vertical axis represents the AUC value of models. **(D)** Five hub genes identified in both COVID-19 and IS from common IDEGs using LASSO logistic regression algorithms.

To further acquire which immune genes were significantly altered and associated with the biological mechanism of COVID-19 and IS, venn diagram analysis was performed between DEGs and IRGs, and 19 common genes were exacted (**Figure 4A**). We further applied the LASSO regression analysis for these genes to screen the gene expression signatures of COVID-19 and IS (**Figures 4B, C**), and finally got five hub genes shared between two diseases (**Figure 4D**) ——*NCR3* (natural cytotoxicity triggering receptor 3), *OLR1* (oxidized low-density lipoprotein receptor 1), *IL1R2* (interleukin 1 receptor type 2)*, IL18R1* (interleukin 18 receptor 1) and *JAK2* (Janus kinase 2). These hub genes can be potential biomarkers and may provide new therapeutic targets. We further validated expression of hub genes and the sensitivity and accuracy of these genes in diagnosis with GSE37587, GSE46480, GSE157103, GSE196822 datasets for IS and COVID-19 respectively. The gene expression showed consistency in test datasets (**Figure S4**) and the AUC value verified the high prediction ability which reached up to 0.96 of five hub genes by various machine learning methods (**Figure S5**).

We further investigated whether there were drugs that could mitigate the expression of essential immune genes (**Figure S6A**). By searching for DTGs associated with IRGs shared between COVID-19 and IS, we identified eight DEGs interacting with two known databases of drug targets: *JAK2, ORM1, RNASE2, TNFSF13B, CYBB, EIF2AK2, CD79B and CAMP* (**Figure S6B**). Among five hub genes shared between two diseases, only *JAK2* has drug target information, which might suggest that *JAK2* could play important roles in the treatment of patients with COVID-19 accompanied with IS.

### 3.4 Network of gene-pathway, miRNA-mRNA interaction and TF-mRNA relationship

**Figure 5:**
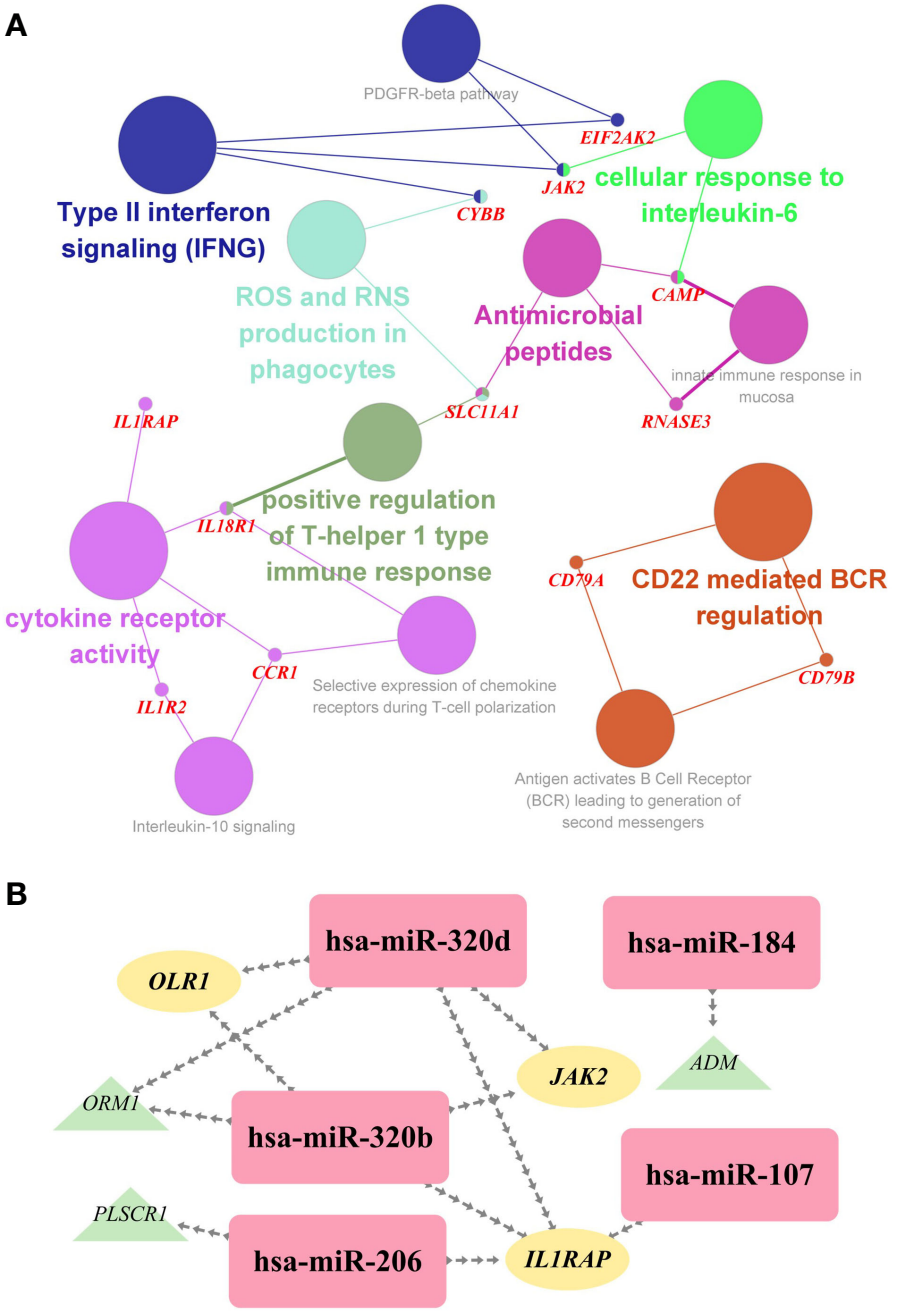
**(A)** The interaction network of GO terms in the common IDEGs presented by the Cytoscape plug-in ClueGO. The most significant term in each group is highlighted. **(B)** The miRNA-mRNA interaction network comprises the common DEGs and corresponding miRNAs. Yellow ellipses represent the hub gene; green triangles represent the common IDEGs; purple diamonds represent the corresponding miRNA targeting.

**Figure 6:**
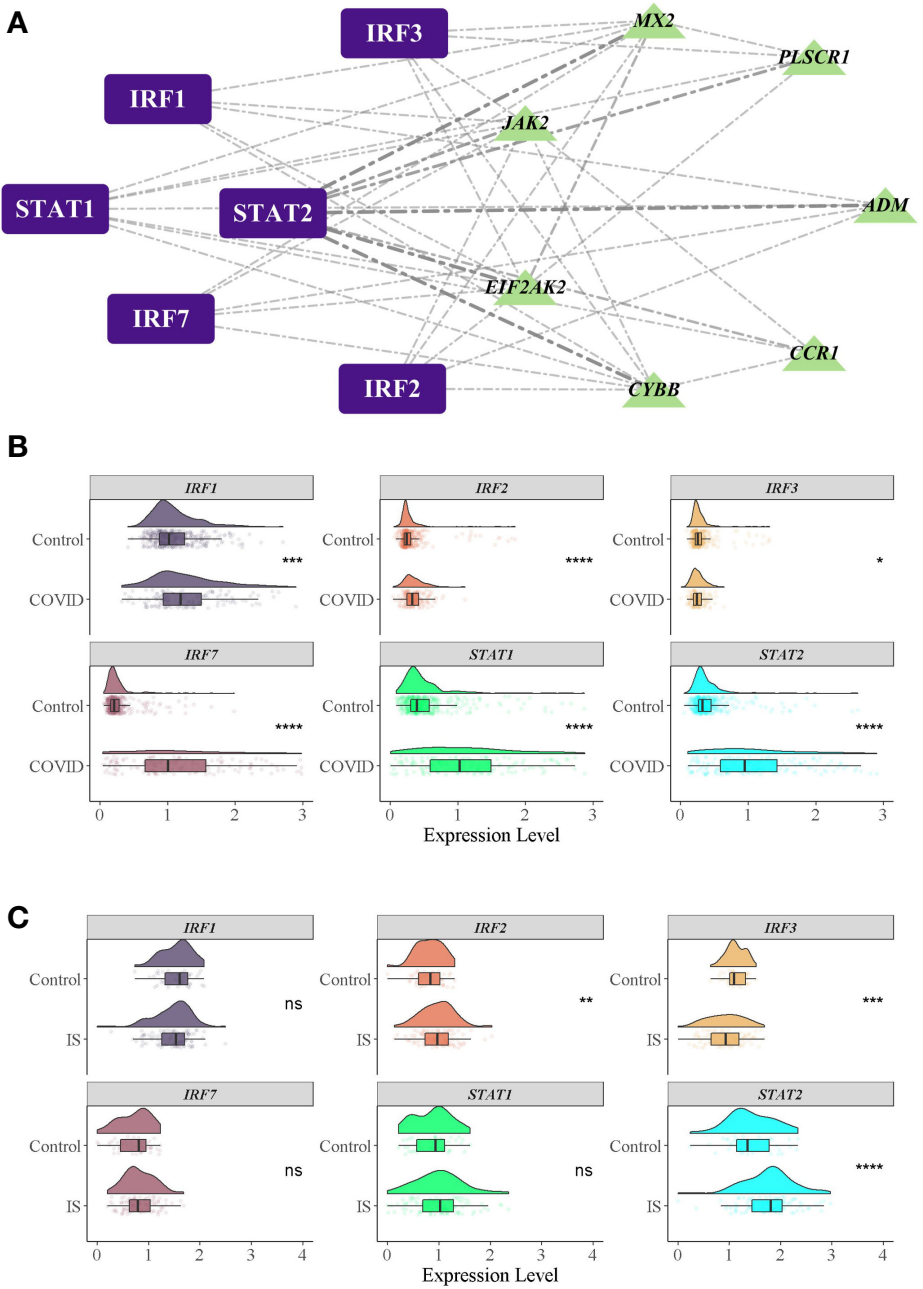
TFs regulatory network and their gene expression profiles in COVID-19/IS. **(A)** Inferring TF regulatory networks. TFs were marked in purple, and the common IDEGs were marked in green. **(B, C)** Gene expression level of TFs in COVID-19 and IS datasets. The comparison of gene expression between patients and controls was applied with t-test. P-value < 0.05 was considered statistically significant. COVID-19, Corona virus disease 2019; IS, ischemic stroke. ^*^:P < 0.05; ^**^:P < 0.01; ^***^:P < 0.001; ^****^:P < 0.0001.

To investigate the biological relationship of immune-related genes, a network of common IDEGs and GO-BP interaction was constructed using ClueGO Plug-in of Cytoscape software. Gene-pathway network reflected that six immune-related biological pathways interacted with these common IDEGs: IFN-γ signaling, cellular response to interleukin 6, antimicrobial peptides, cytokine receptor activity, positive regulation of Th1 immune response and CD22 mediated BCR regulation (**Figure 5A**). With HMDD, TissueAtlas, and ENCORI database, five miRNAs were predicted to interact with the common IDEGs (**Figure 5B**). Based on the RcisTarget package, we found six possible TFs regulating the expression of these common IDEGs (**Figure 6A**), three TFs differentially expressed in the peripheral blood of COVID-19 and IS patients (**Figures 6B, C**). Mainly, *IRF3* expression was down-regulation in two diseases, while *IRF2* and *STAT2* were up-regulated.

### 3.5 Immune changes

**Figure 7:**
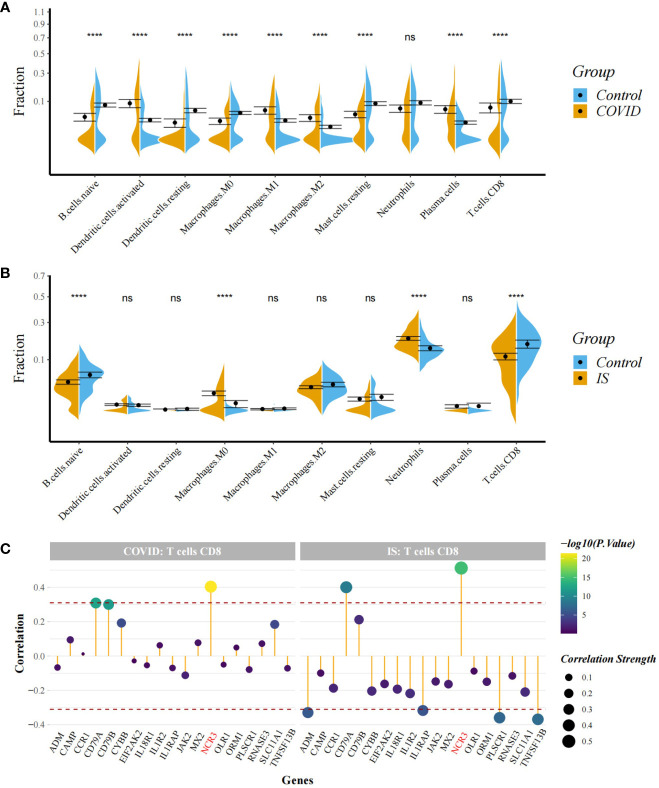
**(A, B)** Comparison of proportion of immune cells between COVID-19/IS and controls with t-test. Analyses were performed using CIBERSORT. ****:P < 0.0001 **(C)** Spearman Correlation Analysis between the gene expression of common IDEGs and CD8^+^ T cell proportion. The red dashed lines represent +0.3 and -0.3. "ns" means P value > 0.05, representing no difference between the case and control.

**Figure 8:**
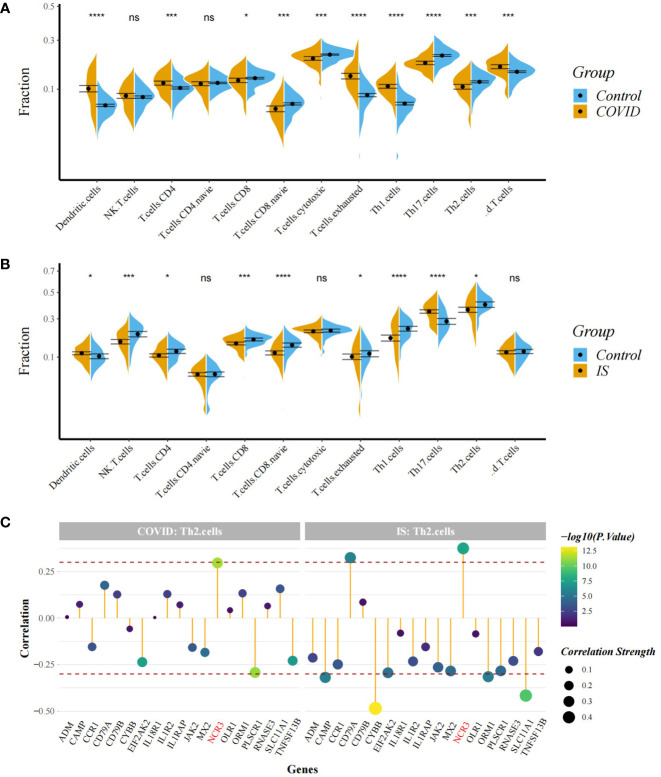
**(A, B)** Comparison of proportion of immune cells between COVID-19/IS and controls with t-test. Analyses were performed using ImmuneCellAI. *:P < 0.05; ***:P < 0.001; ****:P < 0.0001 **(C)** Spearman Correlation Analysis between gene expression of the common IDEGs and Th2 cell proportion. The red dashed lines represent +0.3 and -0.3. "ns" means P value > 0.05, representing no difference between the case and control.

To explore the profile of immune cell infiltration, we applied the CIBERSORT classification algorithm to demonstrate changes in the immune cells in COVID-19 and IS. We found that the proportions of CD8^+^ T cells and naive B cells significantly decreased in both COVID-19 and IS patients compared with healthy controls (**Figures 7A, B**). We further applied ImmuCellAI to focus on the abundance variation of T-cell subpopulations and found that CD8^+^ naive T cells and T helper 2 cell were obviously less enriched in COVID-19 and IS patients (**Figures 8A, B**).

Correlation analysis showed that *NCR3* was positively associated with the change of CD8+ T cells and T helper 2 cell in both COVID-19 and IS patients (r > 0.3, P < 0.001) which indicating a close relationship between hub genes and the profile of immune cell variation (**Figures 7C**, **8C**).

## 4 Discussion

In this study, we analyzed the transcriptome profiles of COVID-19 and IS patients using eight datasets from GEO consisting of 1191 samples to search for common mechanism and molecular biomarkers in COVID-19 and IS. Results demonstrated that immune-related genes and immune cells are crucial in the shared pathogenesis between them. Hub genes, candidate drugs, targeting microRNAs and transcription factors were further analyzed for those immune-related genes shared between two diseases.

Through bioinformatic analyses, we revealed a total of 140 common DEGs shared by COVID-19 and IS. To illustrate the unique biological interpretation of these common DEGs, two datasets, which contain Rheumatoid Arthritis (RA) patients and Sepsis patients separately, were included as the negative controls. As shown in **Figure. S7**, the overlap ratio of COVID and IS (140/537) is significantly larger than that (23/274) of COVID and RA (P value <.001, κ (2) test with Yates’ continuity correction) as well as that (64/827) of COVID and Sepsis (P value <.001, κ (2) test with Yates’ continuity correction). Pathways analysis showed that either the overlapping genes between COVID-19 and Sepsis or between COVID-19 and RA did not enrich in the immune-related pathways (**Figure S8**).

GO and DO enrichment and GSEA analysis were further conducted for these common DEGs. For biological processes, immune-related pathways were outlined, and brain injury in COVID-19 is associated with dysregulated innate and adaptive immune responses. According to the cellular component, the top GO terms are cytoplasmic vesicle lumen, secretory granule lumen and inflammasome complex. The role of NLRP3 inflammasome in stroke was determined *via* various *in vitro* and *in vivo* research, which the viroporins of SARS-CoV2 can activate (37, 38). As expected, top 10 KEGG pathways include Corona Virus Disease-19, lipid and atherosclerosis, complement and coagulation cascades, NOD-like receptor, NF-κB and B cell receptor signaling. Meanwhile, results from the Reactome pathway show the most interacted gene pathways are neutrophil degranulation, interferon α/β signaling, antigen activates BCR leading to generation of second messengers and defects of contact CAS and KKS. Neutrophil degranulation can facilitate a variety of proinflammatory effects, such as cytokine release and fibrin and/or microthrombus formation (39).

For establishing immune-related relationships according to COVID-19 and IS, 19 common immune-related DEGs (IDEGs) were identified. The rest of the research study is continued with the analysis of LASSO regression analysis, gene-pathway interactions, TF/miRNA regulatory network and candidate drug detection.

To get more robust immune-related biomarkers in COVID-19 and IS, LASSO regression analysis was employed to develop gene expression signatures for two diseases. From the 19 common IDEGs, five hub genes were ultimately identified as gene expression signatures to predict disease. Meta-analysis showed that *OLR1* is associated with atherosclerosis and contributes to the susceptibility risk of ischemic stroke (40). Moreover, the proportion of lectin-like OLR1-expressing immature neutrophils is positively correlated with cytokine storm and thrombosis in COVID-19 patients (41). From proteome and transcriptome perspective, several independent cohort studies demonstrated the differential expression of interleukins in COVID-19 and IS patients, including *IL1R2* and *IL18R1 (* 42). JAK1/2 signaling pathway, whose activation contributed to neuronal damage under cerebral ischemic conditions, was critically associated with SARS-CoV-2-induced hypercytokinemia and inflammation (43, 44). The relationship between *NCR3* and COVID-19/IS is currently unclear, although rs2857595 variants near *NCR3* seem to be associated with increased risk of noncardioembolic stroke (45).

We next search for the candidate drugs for COVID-19 and IS based on the intersection across four gene sets, DEGs_COVID, DEGs_IS, IRGs and DTGs. Here, we identified eight DEGs, including *JAK2, ORM1, RNASE2, TNFSF13B, CYBB, EIF2AK2, CD79B and CAMP*. More recently, increased interest in JAKi strategies arose for the need of potential treatments for COVID-19, which is implicated in the activation of CD4^+^ and CD8^+^ positive T cells, NK cells and monocytes that cooperate with cytokine storm generated by SARS-COV2 (46, 47). Two molecules are mainly under focus of pharmaceutical industry, baricitinib and ruxolitinib (23, 48). They are both type I inhibitors with rather low half-life and exhibit IC50s of less than 10 nM for JAK2 (23).

Further multi-network analysis was constructed to identify the most significant functional IDEGs and understand the biological characteristics of the proteins. As a hub gene, *JAK2* greatly participated in gene-pathways and TFs/miRNA-mRNA networks. In gene-pathways network, *JAK2* interacted with IFN-γ signaling and cellular response to IL-6 pathways. Hsa-miR-320d and Hsa-miR-320b interacted with *JAK2* In miRNA-mRNA network. The expression of all miR-320 family members was significantly correlated with the severity and progression of SARS-CoV-2 infection, which also modulates cholesterol efflux and atherosclerosis (49, 50). In silico and microarray analysis proved the regulatory relationship between Hsa-miR-320 and *JAK2 (* 51, 52). In TFs-mRNA network, *JAK2* and *STAT2* mediate the signal transduction of more than 50 cytokines and growth factors in many different cell types, which is critical for resisting infection and enforcing barrier functions. JAK2/STAT2 pathway contributes to homocysteine-accelerated macrophage inflammation, adding to the risk for atherosclerosis (46).

Furthermore, we analyzed the distribution of immune cells in COVID-19/IS patients and found that CD8^+^ T cells, CD8^+^ naive T cells, Th2 cells and naive B cells were differentially distributed between the patients and controls, indicating these immune cells are more important in the common immunological foundation of two diseases. CIBERSORT and ImmuCellAI classification algorithm illuminated the similar decreasing trend in CD8^+^ T cells in COVID-19 and IS patients. CD8^+^ T cells are critical for clearance of many viral infections, due to their ability to kill infected cells (53). However, the decreasing absolute number of lymphopenia was continually observed in patients with COVID-19, especially a severe reduction in the frequency of CD8^+^ T cells (53–55). T-cell exhaustion is evidently the primary mechanism underlying immune dysfunction during viral infection (56). Virus antigen-specific CD8^+^ T cells exhibit features of T-cell exhaustion and dysfunction (57), consistent with our findings (**Figure 8A**). In addition, the decreasing number of T and B lymphocytes in IS patients’ peripheral blood seems to be related to the post-stroke immunosuppression condition (9, 12, 58).

Considering that immunity requires the coordinated efforts of IRGs and immune cells, we analyzed the relationships from common IDEGs to CD8^+^ T cells and Th2 cells. We found a significantly positive correlation between *NCR3* and CD8^+^ T cells and Th2 cells, in both COVID-19 and IS patients. Researchers identified a unique CD8^+^ T-cell cluster expressing innate-like NCR3 protein in healthy donors and patients with viral infection (59, 60). This specific cell group provides a potential explanation for the above correlation between *NCR3* and CD8^+^ T cells. However, the precise mechanism behind *NCR3*, CD8^+^ T cells and Th2 cells is not yet clearly understood.

This study has the following limitations. Firstly, this study was conducted based on bioinformatic and correlational analyses, and differences in microarray platforms, blood collection, and RNA extraction methods, statistical methods could produce potential bias for the results. Besides, the datasets used in this study might not be large enough to generate compelling results. More large cohorts of COVID-19 and IS patients are needed, and future cellular or animal experiments are expected to prove accuracy of the results. Therefore, the above findings should be taken with carefulness. Nevertheless, this study provides new insights into the shared pathogenesis behind COVID-19 and IS, suggesting the critical role of immune changes for the onset and development of these two diseases. Of course, in addition to the close relationship between COIVD and IS, emerging evidence illustrated that immunological response interlink COVID-19 with other diseases, such as HIV infection, cardiovascular disease and periodontitis (61–64).

The blood–brain barrier (BBB), consisting of endothelial cells, vascular smooth muscle cells or pericytes, basement membranes, astrocyte end-feet processes, and neuronal projections, is viewed as the dynamic neurovascular unit (NVU) (65). Recent *in vivo* and *in vitro* research has demonstrated that inflammation and immune response damaging the BBB are the main mechanisms behind the initiation and progression of ischemic stroke. For example, numerous studies showed that thrombin could enter the inflammation-damaged BBB, converting fibrin to fibrinogen, promoting thrombosis (66). Thrombin also could bind with their receptors on the endothelial cells, increasing the cytosolic Ca^2+^ concentration further impairing the BBB (67). Our study indicated that occurrence of post-COVID-19 ischemic stroke might be relevant to inflammatory pathways and immune system, which confirmed with current hypothesis of the importance of inflammation in IS.

## Data availability statement

The original contributions presented in the study are included in the article/**Supplementary Material**. Further inquiries can be directed to the corresponding author.

## Author contributions

WL: Methodology. Investigation, Formal Analysis, Visualization, Writing - Original Draft FH: Supervision, Writing - Review & Editing MW: Writing - Original Draft X-ZY: Conceptualization, Funding Acquisition, Methodology, Writing - Review & Editing. All authors contributed to the article and approved the submitted version.
