# Supplementary material for: Integrated bioinformatics analysis identifies shared immune changes between ischemic stroke and COVID 19
Source: Front Immunol. 2023 Mar 8;14:1102281. doi: 10.3389/fimmu.2023.1102281 (PMC10030956; doi:10.3389/fimmu.2023.1102281)
Supplement: Supplementary file 1 [file DataSheet_1.docx]

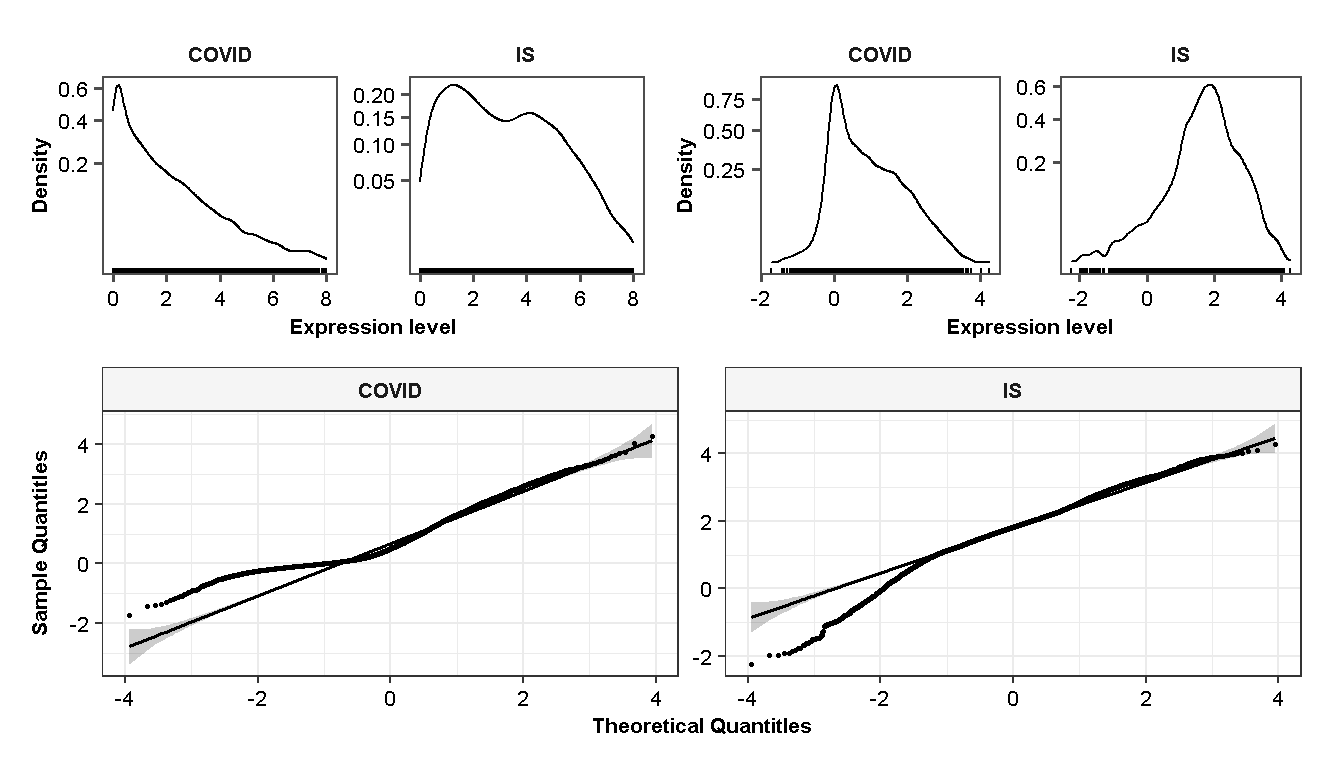


**A**

**B**

**C**

s

**Supplementary Figure 1.** Normalization and correction of datasets used in this study. (A, B) The density plots of COVID and IS datasets before (A) and after (B) normalization and removing batch-effects. (C) The Q-Q plot of the two datasets after correction.


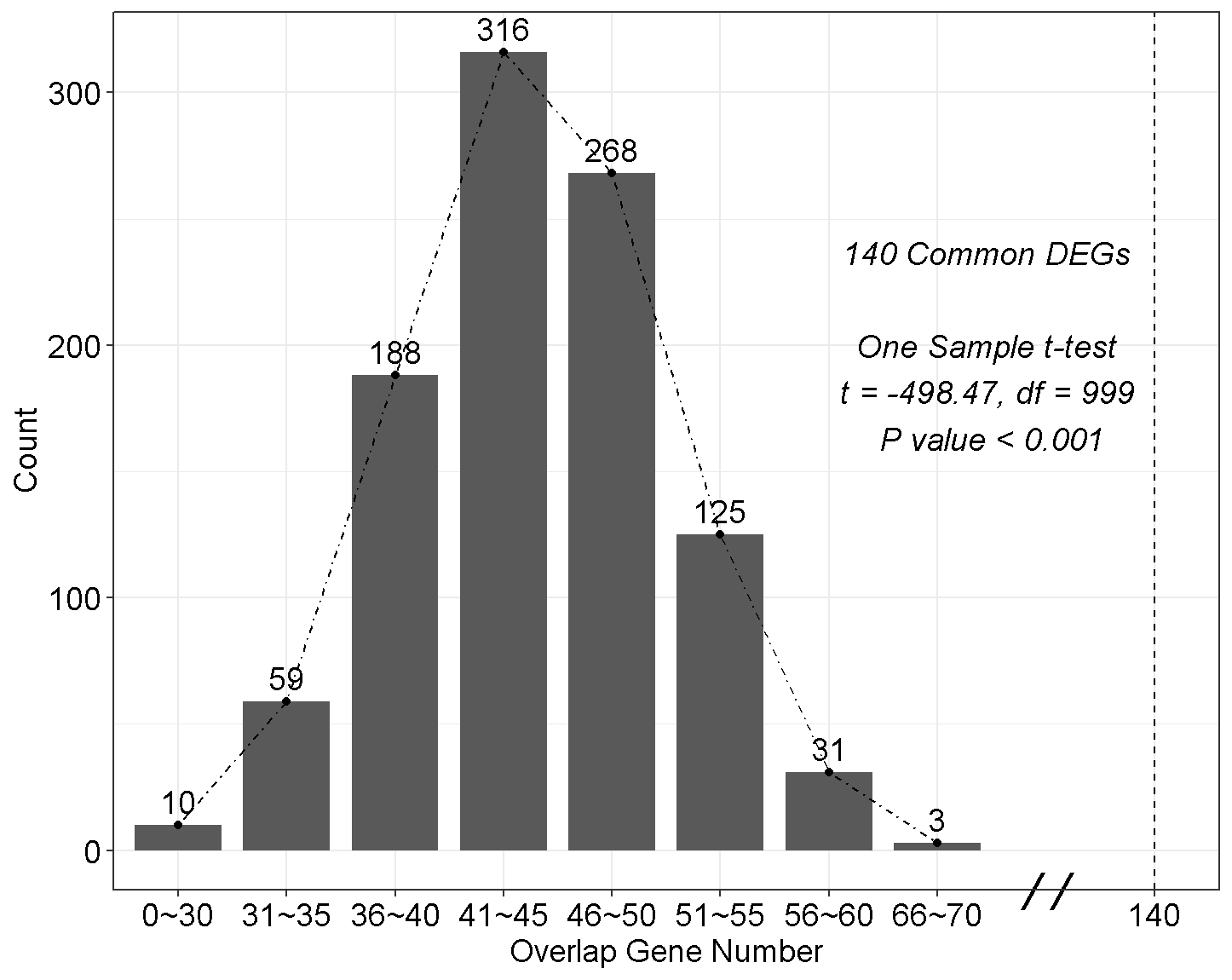
**Supplementary Figure 2.** Simulated distribution of gene number overlapped between COVID and IS. 537 and 1427 genes were randomly selected from IS and COVID datasets respectively for 1000 times and interaction were permutated. The real data was represented with the dotted line.

**Supplementary Figure 3.** DO enrichment analysis. Chord diagram showed the correlation between diseases and common DEGs, with different colors corresponding to different DO terms.

**Supplement Figure 4.** Gene expression level of the hub genes in COVID/IS patients and healthy controls in validated datasets. *:P < 0.05; **:P < 0.01; ***:P < 0.001; ****:P < 0.0001


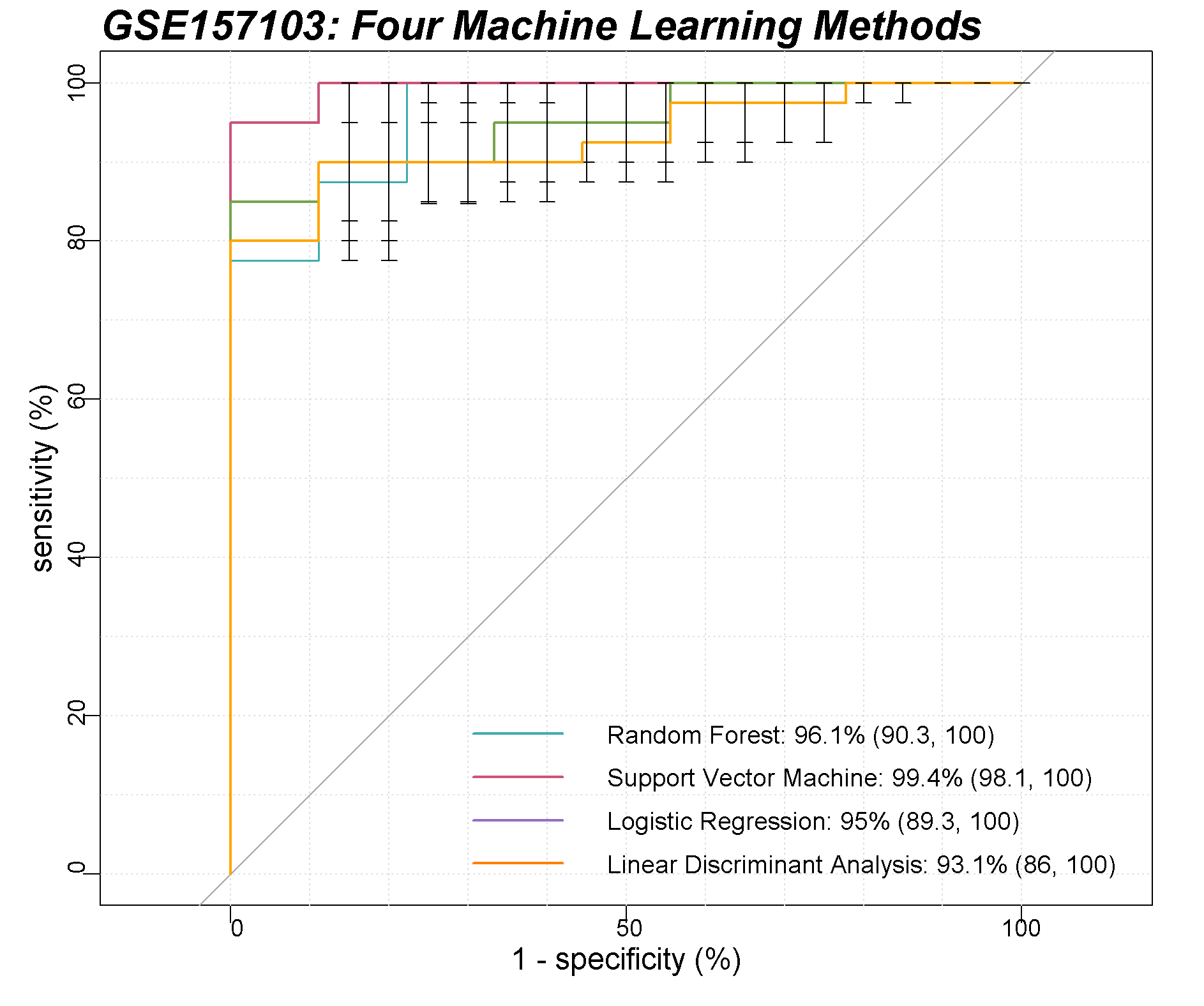

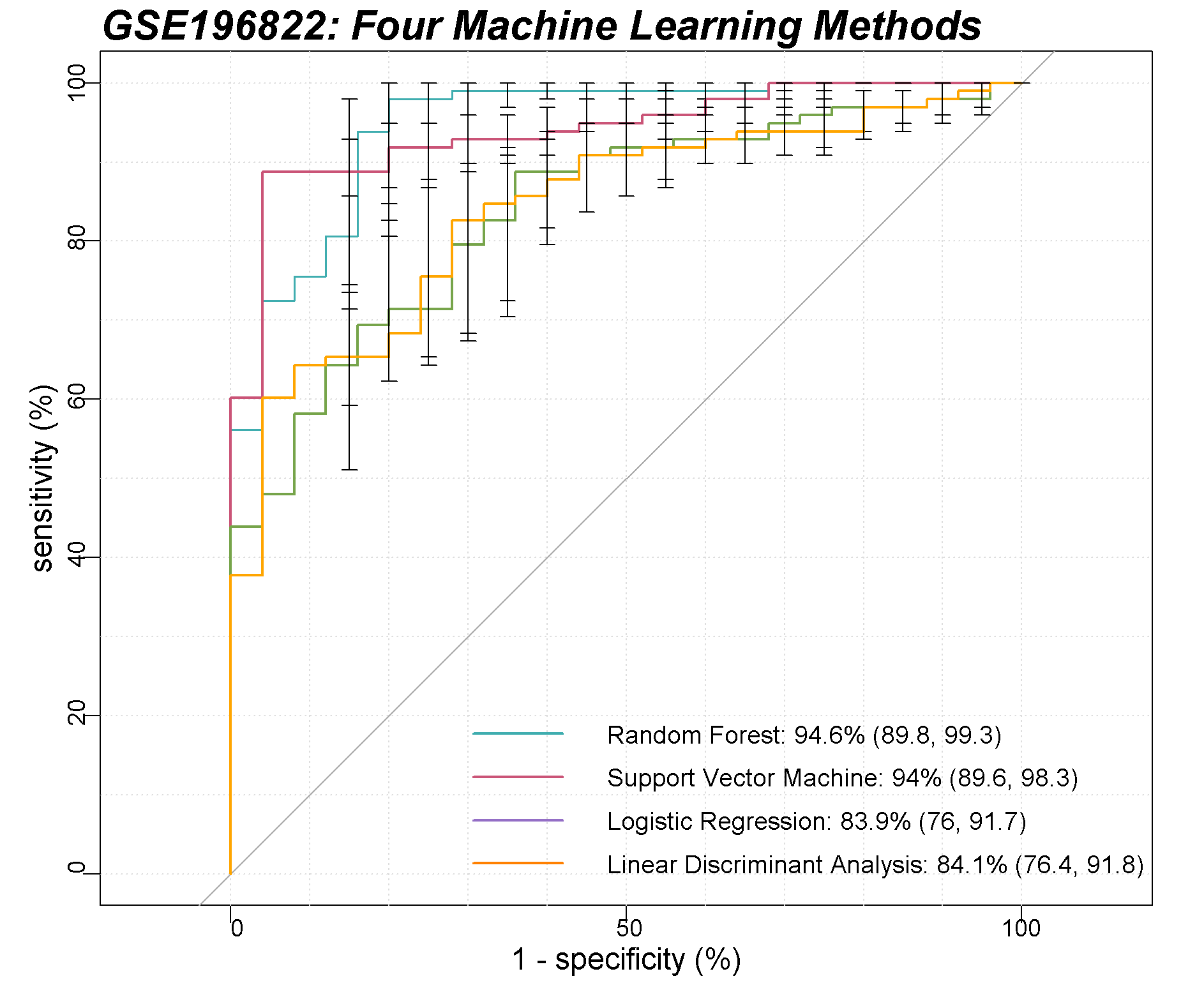

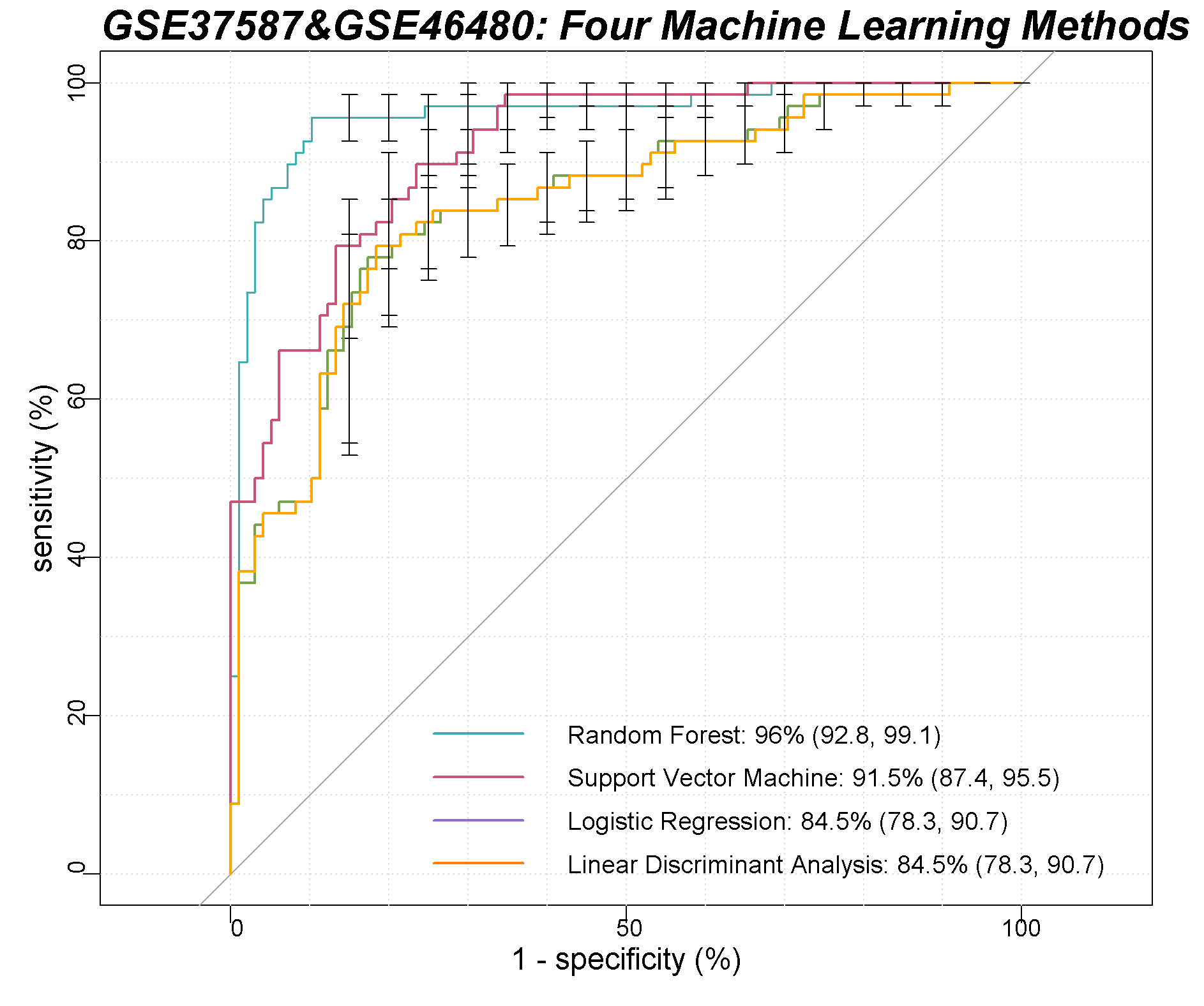


**Supplement Figure 5.** The ROC curve of models of hub genes using various data-modeling methods (Random Forest, SVM, logistic regression, linear discriminant analysis).

**Supplement Figure 6.** (A) Venn diagram shows the drug targets obtained by the four gene sets. (B) The relationship between candidate drugs and its drug targets was depicted in the bar plot.


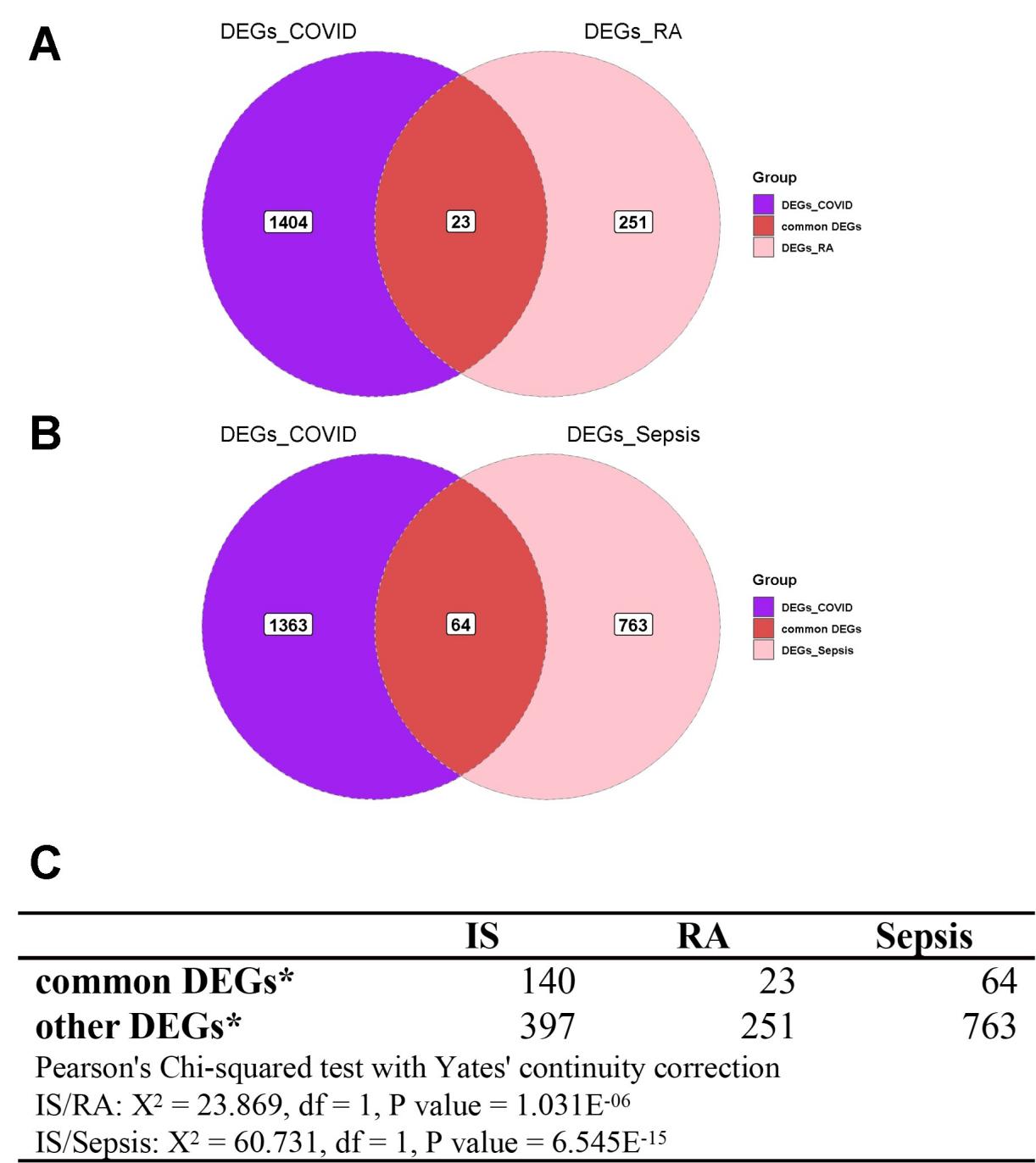


**Supplement Figure 7.** Comparison of common DEGs of COVID-19 and RA/sepsis with COVID-19 and IS. Note: common DEGs* means DEGs shared between IS/RA/Sepsis DEGs with COVID-19 DEGs. Other DEGs* means DEGs only found in IS/RA/Sepsis DEGs.

**Supplement Figure 8.** Gene set enrichment analysis of the the overlapping genes between COVID-19 and RA (A) and COVID-19 and sepsis (B).
